# A Method for En Face OCT Imaging of Subretinal Fluid in Age-Related Macular Degeneration

**DOI:** 10.1155/2014/720243

**Published:** 2014-10-13

**Authors:** Fatimah Mohammad, Justin Wanek, Ruth Zelkha, Jennifer I. Lim, Judy Chen, Mahnaz Shahidi

**Affiliations:** Department of Ophthalmology and Visual Sciences, University of Illinois at Chicago, 1855 W. Taylor Street, Chicago, IL 60612, USA

## Abstract

*Purpose*. The purpose of the study is to report a method for en face imaging of subretinal fluid (SRF) due to age-related macular degeneration (AMD) based on spectral domain optical coherence tomography (SDOCT). *Methods*. High density SDOCT imaging was performed at two visits in 4 subjects with neovascular AMD and one healthy subject. En face OCT images of a retinal layer anterior to the retinal pigment epithelium were generated. Validity, repeatability, and utility of the method were established. *Results*. En face OCT images generated by manual and automatic segmentation were nearly indistinguishable and displayed similar regions of SRF. En face OCT images displayed uniform intensities and similar retinal vascular patterns in a healthy subject, while the size and appearance of a hypopigmented fibrotic scar in an AMD subject were similar at 2 visits. In AMD subjects, dark regions on en face OCT images corresponded to reduced or absent light reflectance due to SRF. On en face OCT images, a decrease in SRF areas with treatment was demonstrated and this corresponded with a reduction in the central subfield retinal thickness. *Conclusion*. En face OCT imaging is a promising tool for visualization and monitoring of SRF area due to disease progression and treatment.

## 1. Introduction

Age-related macular degeneration (AMD) is the leading cause of vision loss in older Americans. A common clinical manifestation of neovascular AMD is accumulation of subretinal fluid (SRF) caused by leakage from choroidal neovascularization (CNV). Currently, intravitreal injection of an antivascular endothelial growth factor agent is the standard of care treatment for CNV. This treatment has resulted in less morbidity and improvement of visual acuity in a sizeable proportion of patients [1, 2]. Spectral domain optical coherence tomography (SDOCT) is used to help guide clinical management of neovascular AMD patients by providing a cross-sectional view of the retinal depth, visualization of SRF, and quantitative measurement of central subfield thickness (CST). Automated techniques based on OCT B-scans or volume data have been developed to identify and measure intraretinal and subretinal fluid [3–7]. Due to recent improvements in OCT technology that allow rapid acquisition of multiple B-scans and dense raster scanning, en face OCT imaging has emerged as a new imaging modality for visualization of retinal structure [8–18]. Recently, en face OCT imaging of the choroid [19, 20] and outer retinal pathologies due to AMD [8, 17, 21, 22] have been demonstrated. The purpose of the current study is to report a method of en face OCT imaging for visualization and monitoring of the spatial extent of SRF in neovascular AMD.

## 2. Methods

High density (HD) SDOCT imaging was performed using a commercial OCT instrument (Spectralis, Heidelberg, Germany) at two visits in 4 subjects with neovascular AMD and one healthy subject. Inclusion criteria for AMD subjects were the presence of subretinal pathologies consisting of SRF that required treatment or subfoveal scars. Subjects with poor fixation or media opacities that affected SDOCT image quality were excluded. HD SDOCT imaging consisted of 145 raster B-scans acquired over a 15° × 15° retinal area centered on the fovea, with 768 A-scans in each B-scan. The SDOCT volume was acquired while the retinal tracking feature of the instrument properly aligned the location of each B-scan on the retina by accounting for eye motion. The axial depth resolution of each B-scan was 3.9 *μ*m. Total retinal thickness maps were generated from the HD SDOCT volume using the instrument's software and the CST was recorded.

From the HD SDOCT volume scans, the depth location of the retinal pigment epithelium (RPE) was automatically identified by smoothing each A-scan using a moving average filter and then detecting the two highest intensity peaks in the A-scan that corresponded to the nerve fiber layer and RPE. The RPE was initially identified as the depth location of the posterior intensity peak of the filtered A-scan. All depth locations were then smoothed using median filtering, thereby generating an estimate of the RPE position in each B-scan. The RPE position was further refined by identifying the maximum intensity pixel in each A-scan located within 10 pixels of this estimate. These locations were again smoothed using a median filter, thereby defining the RPE topography in each B-scan. A layer (~30 microns thick) at a distance of 39 microns (approximately equal to the length of the photoreceptor outer segments) was extracted anterior to the RPE, which was irregular and/or elevated. This approach of en face imaging at the anatomical depth of the photoreceptor inner segment ellipsoid (ISe) was adopted to detect the largest SRF area as compared to more anterior retinal locations and suitably assess the effect of fluid accumulation on photoreceptor function. The mean intensity of the layer was stored in consecutive rows of the en face OCT image, thereby displaying regions with reduced or absent light reflectance due to SRF in an en face manner. By detecting the location of the RPE and extracting an anterior layer in each B-scan, differences in the axial position of the retina in the OCT volume were automatically accounted for in the generated en face OCT image.

Manual segmentation was performed to validate the automated segmentation method for enface image generation. Since manual segmentation of the SDOCT volume was laborious and required excessive time due to the large number of B-scans, manual segmentation was only performed on 2 sets of 145 B-scan images in an AMD subject by one observer. For manual segmentation, the RPE profile was outlined in each B-scan of the HD SDOCT volume data set using a commercial software package (Image J). An automated software algorithm detected the RPE profile and then averaged intensity data from a retinal layer anterior to the RPE to generate an en face OCT image, similar to the automated procedures described above.

## 3. Results

**Figure 1:**
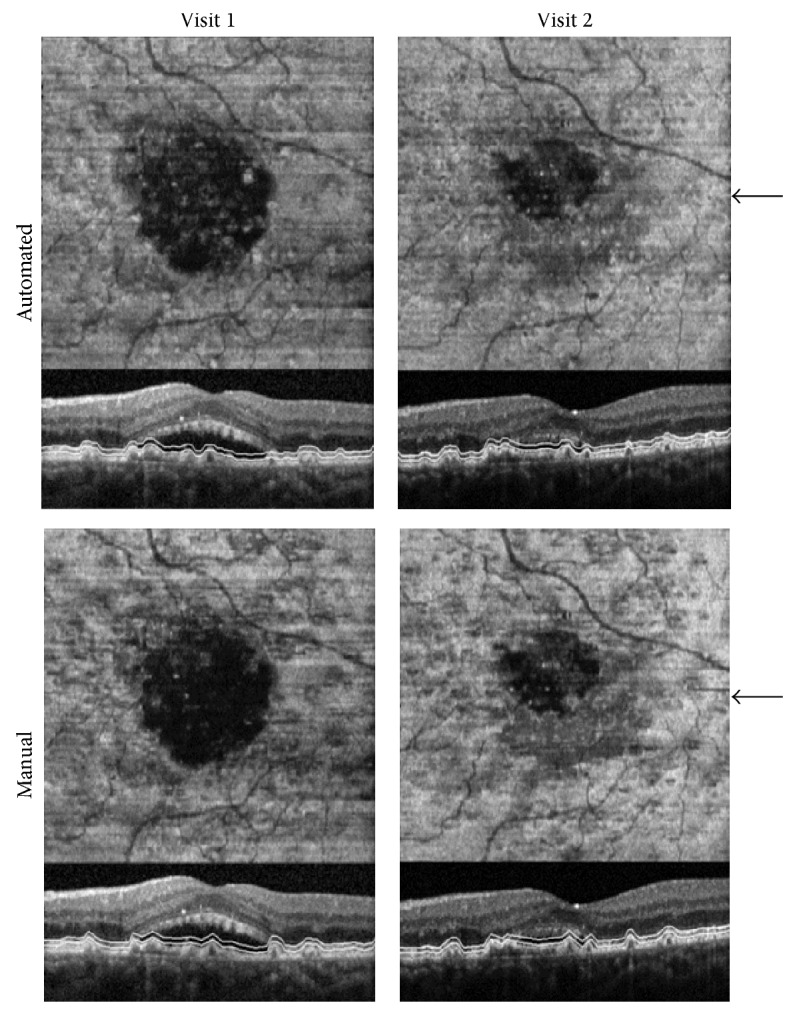
En face OCT images generated by automated and manual segmentation at two visits in a subject treated for neovascular AMD. Arrows indicate the locations of the HD SDOCT B-scans shown below en face OCT images. White lines on B-scans indicate the segmented layer depth location of en face OCT images. Dark regions correspond to SRF and appear nearly identical in en face OCT images generated manually and automatically.

En face OCT image generation was validated by comparing images obtained by automated and manual segmentation. HD SDOCT imaging was performed at 2 visits (5 months apart) in the left eye of a 68-year-old male with a history of neovascular AMD and SRF. The subject received treatment of intravitreal injections of ranibizumab (Lucentis, Genentech, Roche) according to clinical protocol. En face OCT images generated by manual and automated segmentation are shown in Figure 1. On en face OCT images, irregularly shaped dark regions were observed corresponding to SRF. The area of the dark region was reduced after treatment, indicating a reduction in the SRF. The shape and area of SRF regions were nearly identical on en face OCT images generated manually and automatically.

**Figure 2:**
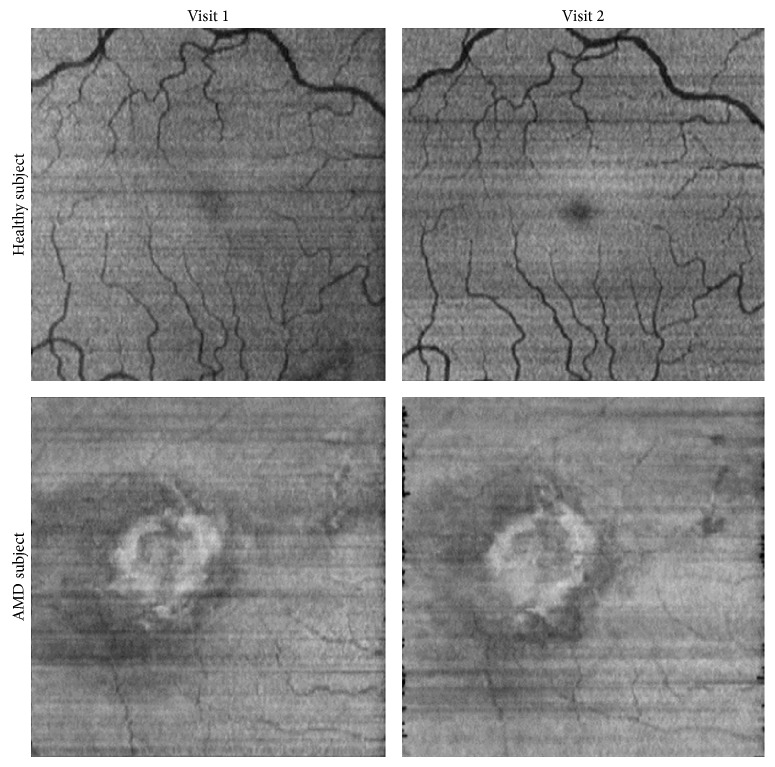
En face OCT images generated at two visits in a visually normal subject and an AMD subject. The retinal vascular pattern and hypopigmented fibrotic scar appear similar on en face OCT images obtained at the 2 visits.

The consistency and repeatability of automated en face OCT image generation were demonstrated by comparing images generated at 2 visits in a visually normal subject and an AMD subject with a fibrotic scar in which minimal retinal changes were anticipated. Examples of en face OCT images obtained at 2 visits (8 months apart) in the right eye of the 30-year-old visually normal male are shown in Figure 2. The appearance of en face OCT images obtained at the 2 visits was similar. Both images displayed shadows of the overlying retinal vasculature and had uniform intensities. En face OCT images obtained at 2 visits (1 month apart) in the right eye of the 80-year-old female diagnosed with a subfoveal scar and neovascular AMD are shown in Figure 2. On en face OCT images, bright regions were visible due to light scatter from the hypopigmented fibrotic scar. As expected, this region was similar between visits indicating minimal change in the scar extent.

**Figure 3:**
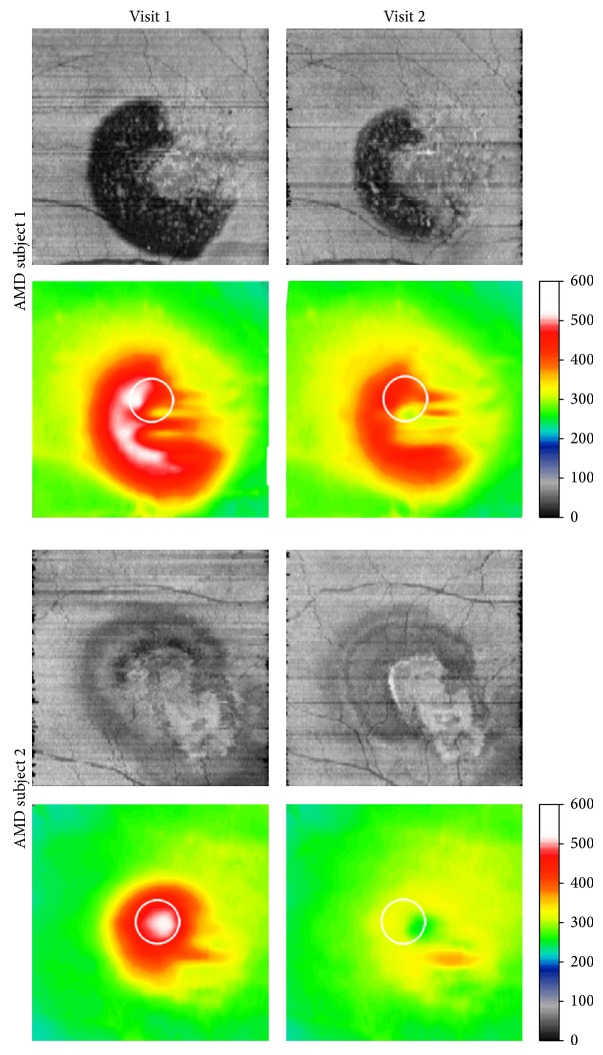
En face OCT images generated at two visits in 2 subjects treated for neovascular AMD. Corresponding retinal thickness maps are shown below en face OCT images. White circles indicate the central subfield location and color bars denote thickness in microns. SRF regions and central subfield thickness were reduced with treatment.

The utility of en face OCT imaging for detection of changes in SRF was demonstrated by comparing en face images obtained during the course of intravitreal injection treatment of 2 AMD subjects at 2 visits. Examples of en face OCT images and retinal thickness maps obtained at 2 visits in the left eye of an 81-year-old female with recurrent CNV and SRF are shown in Figure 3. On en face OCT images, large irregularly shaped dark regions were observed corresponding to SRF. The area of SRF and CST were reduced with treatment. Examples of en face OCT images and retinal thickness maps obtained at 2 visits in the right eye of a 74-year-old male with a history of CNV are shown in Figure 3. On en face OCT images, a bright region corresponding to RPE hypopigmentation was visualized and the boundary of this region was better defined after treatment. Superior to the bright region, en face OCT images displayed regions with reduced intensity corresponding to SRF. The area of SRF and CST were reduced with treatment.

## 4. Discussion

In neovascular AMD, the presence and progressive increase of SRF can adversely affect visual function. Improvement in visual acuity by reduction in SRF is a desired outcome of currently available treatments of neovascular AMD. The current study reports an en face OCT imaging method for detecting and monitoring changes in the spatial extent of SRF that may be beneficial for monitoring pathologies and guiding treatment outcomes.

En face OCT imaging for assessment of SRF has several advantages. Since en face OCT images are reconstructed from HD SDOCT B-scans with high depth discrimination, they have high contrast and thus offer an accurate and efficient method for delineating the spatial extent of SRF. In addition, en face OCT images can be easily combined or registered with data acquired from other imaging modalities. Currently, a qualitative assessment of changes in SRF extent requires viewing 2 sets of multiple B-scans. This subjective method does not provide a quantitative and objective measure for monitoring disease progression and evaluating response to treatment. In contrast, on en face OCT images, the spatial extent of SRF can easily and clearly be visualized and quantitatively measured at each visit. Furthermore, SRF visualized on en face OCT images are likely related to visual dysfunction [23–25], since they were generated at a retinal depth that was in close proximity to the photoreceptor cells and captured the largest spatial extent of fluid accumulation.

The reported automated method of en face OCT image generation was shown to reliably image the extent of SRF and the validity and repeatability of en face OCT images were established. Additionally, the utility of en face OCT images for visualizing SRF and changes in SRF with treatment was demonstrated. Dark regions on en face OCT images corresponded to absent or reduced light reflectance due to SRF. These regions were irregularly shaped and variable in size. A decrease in the area of these regions indicated a reduction in SRF, with a corresponding decrease in the CST with treatment. The current study was limited by the small sample size. Future studies in a larger population are warranted to further evaluate the utility of the method and establish its advantages as compared to en face OCT images generated by commercially available instruments. Additionally, light scatter from overlying retinal pathologies may affect light reflectance detected from underlying retinal layers. However, compiled en face OCT images generated at multiple retinal depths may be useful in discriminating SRF from shadowing of pathologies.

## 5. Conclusion

In summary, an en face OCT imaging method for visualization and monitoring of SRF in neovascular AMD subjects was demonstrated. This method is of potential value for monitoring alterations in SRF area due to disease progression and treatment.
